# Mortality trends of gallbladder cancer in middle-aged and older adults in the United States: a CDC WONDER analysis from 1999 to 2023

**DOI:** 10.3389/fonc.2026.1881189

**Published:** 2026-08-12

**Authors:** Qiuxuan Yang, Xu Chen, Yao Xiao, Qun Xiao, Xiaoli Li

**Affiliations:** 1 Department of General Surgery, Xiangya Hospital, Central South University, Changsha, China; 2 National Clinical Research Center for Geriatric Disorders, Xiangya Hospital, Central South University, Changsha, China; 3 International Joint Research Center of Minimally Invasive Endoscopic Technology Equipment & Standards, Changsha, China; 4 Department of Neurosurgery, Xiangya Hospital, Central South University, Changsha, China

**Keywords:** CDC WONDER, epidemiology, gallbladder cancer, mortality, racial disparity, sex disparity

## Abstract

**Background:**

Gallbladder cancer (GBC) is a rare but highly lethal malignancy, often diagnosed at advanced stages due to the absence of early symptoms. Although its incidence is relatively low in the United States, marked demographic and geographic disparities persist, and long-term national mortality trends remain insufficiently characterized.

**Methods:**

We analyzed gallbladder cancer·–related deaths (ICD-10 code C23) among U.S. adults aged 45 years and older from 1999 to 2023 using the CDC WONDER database. Age-adjusted mortality rates (AAMRs) per 100,000 were calculated. For age-stratified analyses, crude mortality rates were used instead of AAMRs, as age adjustment within predefined strata is inappropriate. Temporal trends were assessed using joinpoint regression to estimate annual percent change (APC) and average annual percent change (AAPC) overall and by sex, age group, race and ethnicity, census region, and urbanization level.

**Results:**

During the study period, 50,938 gallbladder cancer–related deaths were recorded. Overall, the AAMR declined from 2.10 per 100,000 in 1999 to 1.39 per 100,000 in 2023, with significant decreases across multiple time intervals (AAPC = -1.76, 95% CI: -2.16 to -1.36, P ≤ 0.05). Mortality increased markedly with age, and individuals aged 85 years and older consistently exhibited the highest rates. Females had higher mortality rates than males throughout the study period (1.72 vs. 1.01 per 100,000 in 2023), although declining trends were observed in both sexes. Significant reductions in mortality were observed among Hispanic, non-Hispanic White, and non-Hispanic Other populations; however, no significant decline was observed among non-Hispanic Black individuals (AAPC = -0.14, 95% CI: -0.60 to 0.33, P > 0.05). Regionally, mortality was highest in the Northeast and lowest in the South. Mortality rates were consistently higher in metropolitan than in non-metropolitan areas (1.51 vs. 1.48 per 100,000 in 2020).

**Conclusion:**

Although gallbladder cancer mortality among middle-aged and older adults in the United States has declined over the past two decades, this improvement has been uneven. Persistent disparities by age, sex, race and ethnicity, region, and urbanization remain, particularly among non-Hispanic Black populations. These findings underscore the need for targeted and equitable public health strategies to further reduce gallbladder cancer mortality.

## Introduction

Gallbladder cancer (GBC) is the most common malignancy of the biliary tract and is associated with a poor prognosis (1). Many cases are detected incidentally during cholecystectomy performed for symptomatic gallstones (1). Because early clinical manifestations are often nonspecific or absent, diagnosis frequently occurs at advanced stages when therapeutic options are limited. Long-standing inflammation is considered the principal driver of malignant transformation, although additional contributors—including gallbladder polyps, metabolic factors, congenital biliary anomalies, primary sclerosing cholangitis, family history of gallstone disease, chronic bacterial infection, heavy metal exposure, and dietary risks—have been identified (1–3).

Although the global incidence of GBC remains relatively low, substantial geographic and ethnic disparities persist (4). Elevated incidence and mortality have been documented in Indigenous communities throughout the Americas and among certain Asian populations (4, 5). In the United States, the 5-year survival rate is approximately 18 percent, reflecting continued challenges in early detection and treatment (5). Regional patterns may be influenced by environmental exposures, including fungal toxins with carcinogenic potential (6, 7). Sex disparities have been consistently reported, with higher incidence and mortality among females than males (5, 8). Recent evidence also suggests increasing incidence in younger adults and among non-Hispanic Black populations (8–11). These trends raise concern that the burden of GBC in the United States may continue to rise, particularly in susceptible subgroups.

Despite these emerging patterns, limited research has examined long-term U.S. mortality trends for GBC across demographic and geographic strata. Prior studies have focused primarily on incidence or on shorter observation windows, leaving an incomplete understanding of evolving mortality patterns among aging populations. To address this gap, the present study analyzed national mortality data from 1999 to 2023 among adults aged 45 years and older using CDC WONDER records. We assessed temporal trends overall and across sex, age group, race and ethnicity, census region, and urbanization level. A clearer understanding of long-term mortality patterns may help identify disproportionately affected populations and inform targeted and equitable public health strategies to reduce GBC mortality.

## Methods

### Data source

We obtained national mortality data from the Centers for Disease Control and Prevention (CDC) Wide-Ranging Online Data for Epidemiologic Research (WONDER) Underlying Cause of Death files (http://wonder.cdc.gov/). Deaths attributed to gallbladder cancer were identified using the International Classification of Diseases, Tenth Revision (ICD-10) code C23. Age-adjusted mortality rates (AAMRs) were calculated per 100,000 persons and standardized to the 2000 U.S. population. However, age-specific analyses were performed using crude mortality rates rather than age-adjusted mortality rates because age adjustment within predefined age strata is not appropriate.

### Study population and subgroups

The study population included U.S. adults aged 45 years and older who died between 1999 and 2023. This age threshold was selected because gallbladder cancer is exceedingly rare in individuals younger than 45 years; restricting the analysis to middle-aged and older adults ensures the statistical stability of mortality estimates and focuses on the most clinically relevant population. Furthermore, this approach aligns with established methodological frameworks in recent CDC WONDER-based mortality studies utilizing similar age stratifications (12). Mortality counts and rates were stratified by sex, age group, race and ethnicity, census region, and urbanization level. Race and ethnicity were classified as Hispanic, non-Hispanic White, non-Hispanic Black, and non-Hispanic Other. Urbanization was categorized as metropolitan or non-metropolitan based on the National Center for Health Statistics classification (13). It is important to note that due to data availability in the CDC WONDER database, the analysis stratified by urbanization level was restricted to the period from 1999 to 2020, whereas all other analyses extended through 2023.

### Statistical analysis and definition of measures

Temporal trends in AAMRs were quantified using the Joinpoint Regression Program (version 5.0.2, National Cancer Institute, USA). The model allowed detection of up to four trend inflection points within the study period. The optimal number of joinpoints and the best-fitting model were determined using the Monte Carlo permutation test with a significance level of 0.05. For each segment, annual percent change (APC) and its corresponding 95 percent confidence interval (CI) were estimated. The average annual percent change (AAPC) for overall trends was calculated as a weighted mean of segment-specific APCs. Statistical significance was defined as P ≤ 0.05. Data visualization was performed using R software (version 4.5.1).

### Ethics statement

The CDC WONDER database provides publicly available, aggregate-level data without personal identifiers. Accordingly, institutional review board approval was not required for this study.

## Results

### Overall mortality trends

**Figure 1:**
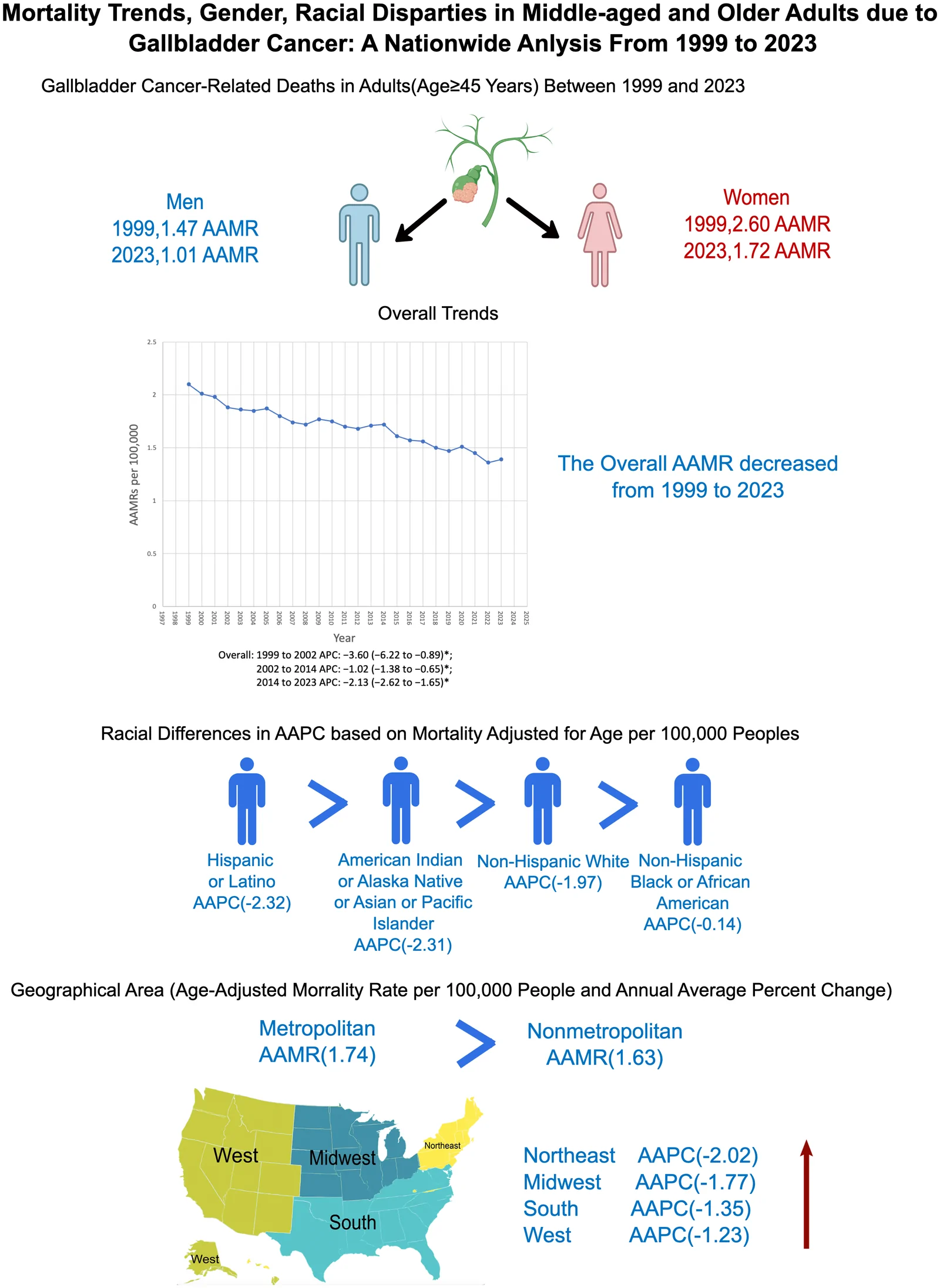
Mortality trends of gallbladder cancer in middle-aged and older adults in the United States: A CDC WONDER analysis from 1999 to 2023.

**Figure 2:**
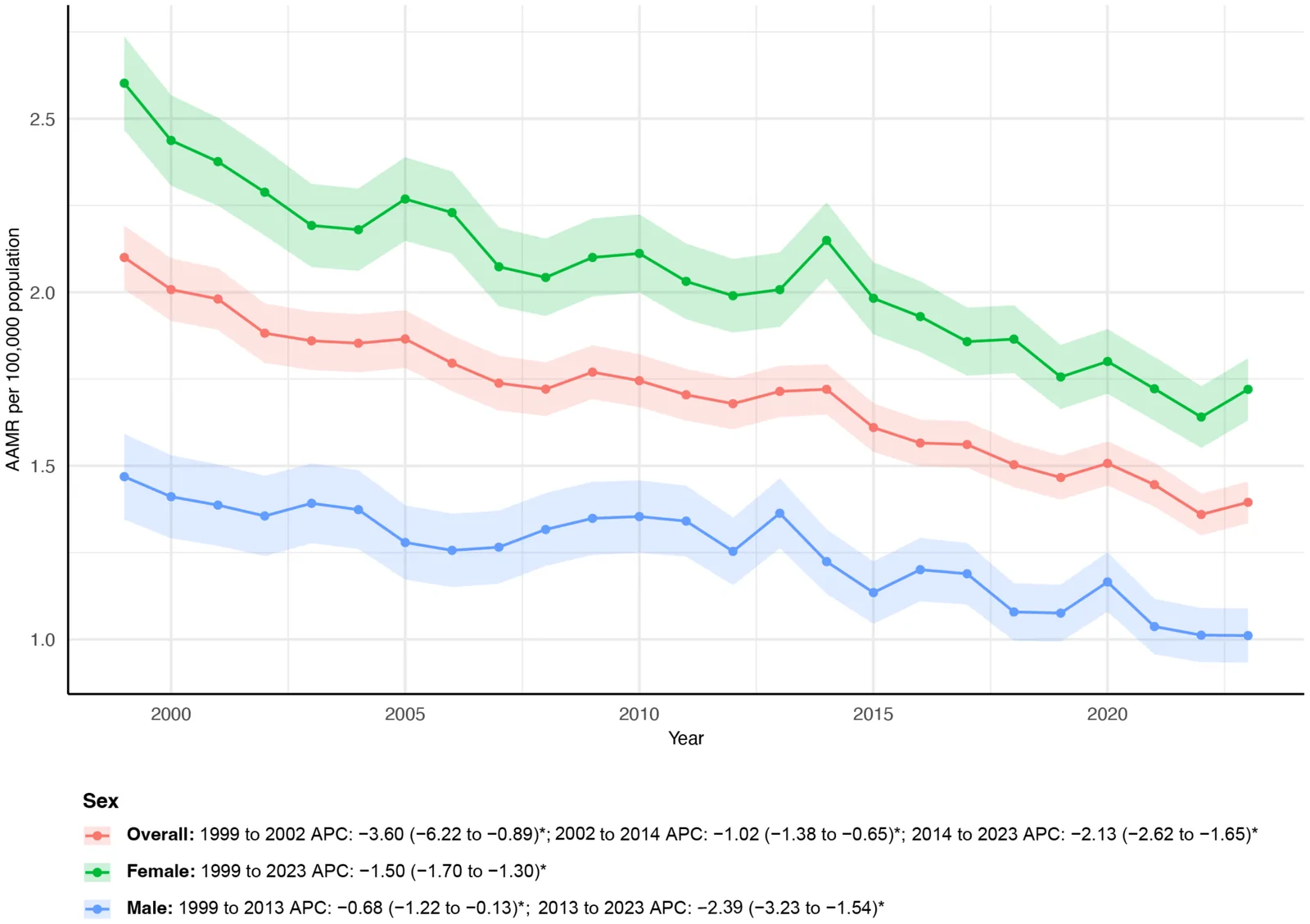
Overall and sex-stratified gallbladder cancer-related age-adjusted mortality rates per 100,000 in middle-aged and older adults in the United States, 1999–2023. *Indicates that the annual percentage change (APC) is significantly different from zero at α = 0.05.

From 1999 to 2023, a total of 50,938 gallbladder cancer–related deaths were recorded among U.S. adults aged 45 years and older. The overall age-adjusted mortality rate (AAMR) declined from 2.10 per 100,000 people in 1999 to 1.39 per 100,000 people in 2023 (**Table 1**). Joinpoint regression identified a significant decrease in mortality across multiple time intervals, including a pronounced decline from 1999 to 2002 (annual percent change [APC]: −3.60; 95% CI: −6.22 to −0.89, P ≤ 0.05), followed by a sustained reduction from 2002 to 2014 (APC: −1.02; 95% CI: −3.18 to −0.65, P ≤ 0.05) and a further accelerated decline from 2014 to 2023 (APC: −2.13; 95% CI: −2.62 to −1.65, P ≤ 0.05) (**Figures 1**, **2**; **Supplementary Tables 1**, **S2**, **S3**).

**Table 1:** Trends in gallbladder cancer mortality, age-adjusted mortality rates and age-specific crude mortality rates in the United States, 1999–2023.

Characteristic	Deaths			AAMR		
	1999	2023	Percent Change (%)	1999 (95% CI)	2023 (95% CI)	AAPC (95% CI)
Overall	2,019	2,134	5.70	2.10 (2.01 to 2.19)	1.39 (1.34 to 1.45)	-1.76 (-2.16 to -1.36) ^a^
Sex						
Female	1,452	1,459	0.48	2.60 (2.47 to 2.74)	1.72 (1.63 to 1.81)	-1.50 (-1.70 to -1.30) ^a^
Male	567	675	19.05	1.47 (1.35 to 1.59)	1.01 (0.93 to 1.09)	-1.39 (-1.84 to -0.95) ^a^
Census region						
Northeast	509	400	-21.41	2.52 (2.30 to 2.74)	1.44 (1.30 to 1.58)	-2.02 (-2.27 to -1.77) ^a^
Midwest	520	460	-11.54	2.30 (2.11 to 2.50)	1.44 (1.31 to 1.57)	-1.77 (-2.10 to -1.44) ^a^
South	607	754	24.22	1.81 (1.67 to 1.96)	1.30 (1.20 to 1.39)	-1.35 (-1.63 to -1.07) ^a^
West	383	520	35.77	2.02 (1.82 to 2.23)	1.53 (1.39 to 1.66)	-1.23 (-2.12 to -0.33) ^a^
Race						
Hispanic	200	353	76.50	4.01 (3.43 to 4.58)	2.12 (1.89 to 2.35)	-2.32 (-2.83 to -1.80) ^a^
NH Black	193	329	70.47	2.44 (2.09 to 2.78)	2.17 (1.92 to 2.41)	-0.14 (-0.60 to 0.33)
NH White	1,548	1,302	-15.89	1.93 (1.84 to 2.03)	1.16 (1.10 to 1.22)	-1.97 (-2.15 to -1.78) ^a^
NH Other	74	148	100.00	2.99 (2.33 to 3.78)	1.40 (1.18 to 1.63)	-2.31 (-2.97 to -1.66) ^a^
Urbanization						
Metropolitan	1,658	1,875	13.09	2.16 (2.05 to 2.26)	1.51 (1.44 to 1.58) ^b^	-1.68 (-2.22 to -1.14) ^a, b^
Nonmetropolitan	361	360	-0.28	2.03 (1.82 to 2.25)	1.48 (1.32 to 1.63) ^b^	-1.61 (-2.05 to -1.17) ^a b^
Age group						
45–54 years	127	123	-3.15	0.35 (0.35 to 0.35) ^c^	0.30 (0.30 to 0.30) ^c^	-0.77 (-1.33 to -0.21) ^a c^
55–64 years	309	334	8.09	1.30 (1.30 to 1.30) ^c^	0.80 (0.80 to 0.80) ^c^	-1.27 (-1.62 to -0.91) ^a c^
65–74 years	552	683	23.73	3.00 (3.00 to 3.00) ^c^	1.97 (1.97 to 1.97) ^c^	-1.64 (-1.92 to -1.36) ^a c^
75–84 years	686	650	-5.25	5.61 (5.61 to 5.61) ^c^	3.54 (3.54 to 3.54) ^c^	-1.80 (-2.31 to -1.29) ^a c^
85+ years	345	344	-0.29	8.31 (8.31 to 8.31) ^c^	5.55 (5.55 to 5.55) ^c^	-1.58 (-1.84 to -1.32) ^a c^

The footnote ^a^indicates statistically significant AAPC values. The footnote ^b^denotes that the AAMR data for 2023 urbanization levels are based on 2020 data, and AAPC calculations are derived from the period between 1999 and 2020. Footnote ^c^indicates that the AAMR and AAPC for age groups are both based on crude mortality rates. AAMR, Age-Adjusted Mortality Rate. AAPC, Annual Average Percent Change. NH, non-Hispanic.

### Sex-specific mortality trends

Throughout the study period, females consistently showed higher AAMRs than males did. Among females, the AAMR declined significantly from 2.60 per 100,000 in 1999 (95% CI: 2.47 to 2.74) to 1.72 per 100,000 in 2023 (95% CI: 1.63 to 1.81), corresponding to an average annual percent change (AAPC) of −1.50 (95% CI: −1.70 to −1.30, P ≤ 0.05). A similar downward trend was observed among males, with AAMRs decreasing from 1.47 per 100,000 (95% CI: 1.35 to 1.59) in 1999 to 1.01 per 100,000 (95% CI: 0.93 to 1.09) in 2023 (AAPC: −1.39; 95% CI: −1.84 to −0.95, P ≤ 0.05).

Temporal analysis revealed differences in the pace of decline by sex. Among females, mortality declined steadily over the entire study period. Among males, a modest decline was observed from 1999 to 2013 (APC: −0.68; 95% CI: −1.22 to −0.13, P ≤ 0.05), followed by a more pronounced decrease from 2013 to 2023 (APC: −2.39; 95% CI: −3.23 to −1.54, P ≤ 0.05) (**Figure 2**; **Supplementary Tables 2**, **S3**).

### Age-specific mortality patterns

When stratified by 10-year age groups, analyses were evaluated using crude mortality rates rather than age-adjusted rates. The largest number of deaths occurred among individuals aged 75–84 years (n = 15,545), followed by those aged 65–74 years (n = 14,377), 85 years and older (n = 8,820), 55–64 years (n = 8,755), and 45–54 years (n = 3,441). Across all study years, crude mortality rates increased markedly with age, with the highest rates consistently observed in the ≥85-year age group.

**Figure 3:**
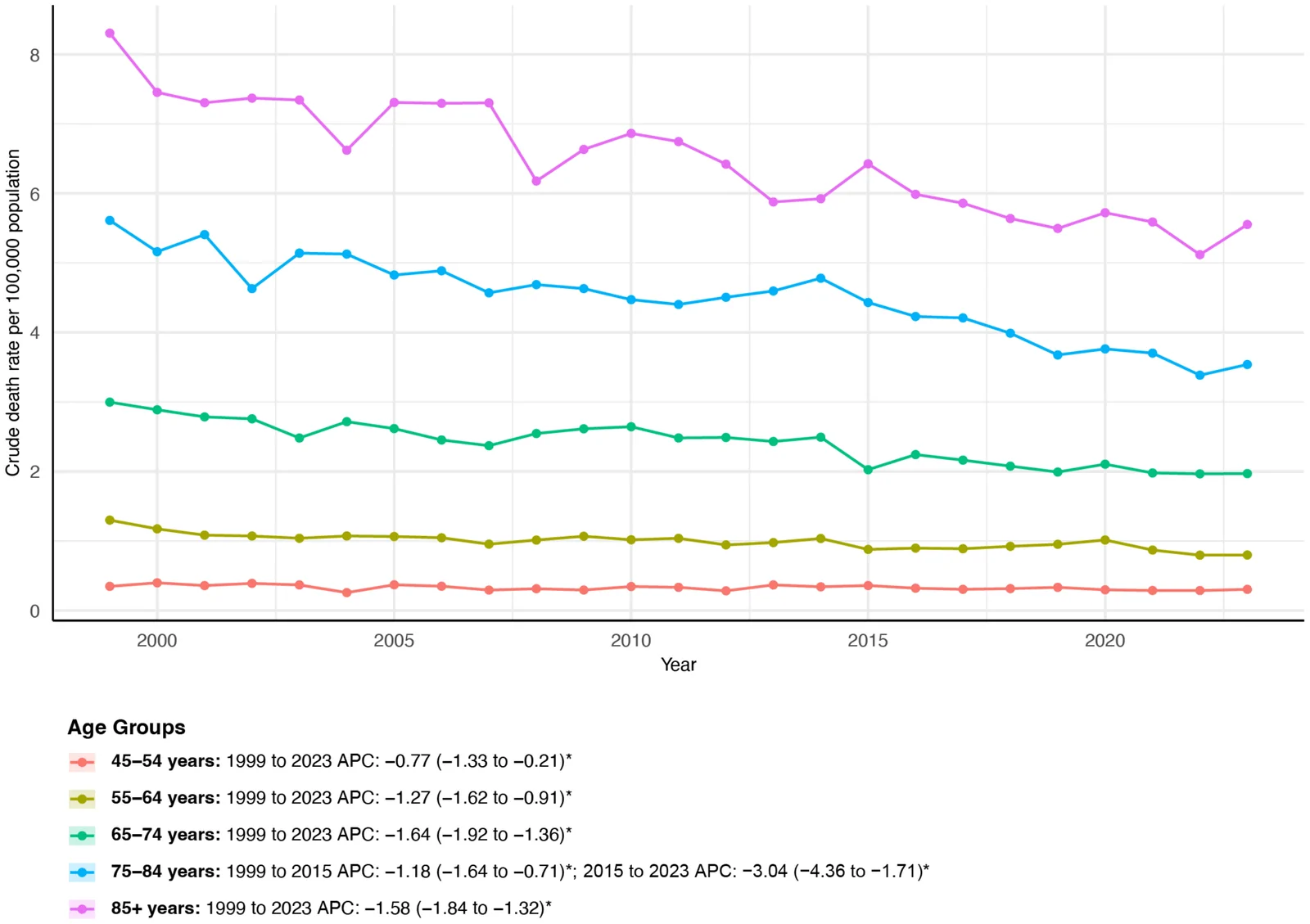
Gallbladder cancer-related crude death rate per 100,000, stratified by age groups in middle-aged and older adults in the United States, 1999–2023. *Indicates that the annual percentage change (APC) is significantly different from zero at α = 0.05.

From 1999 to 2023, all age groups demonstrated significant declines in crude mortality rates. The ≥85-year group showed a sustained decrease over time (APC: −1.58; 95% CI: −1.84 to −1.32, P ≤ 0.05). Among individuals aged 75–84 years, mortality declined modestly from 1999 to 2015 (APC: −1.18; 95% CI: −1.64 to −0.71, P ≤ 0.05), followed by a steeper decline from 2015 to 2023 (APC: −3.04; 95% CI: −4.36 to −1.71, P ≤ 0.05). Consistent downward trends were also observed in the 65–74, 55–64, and 45–54 year age groups (**Figure 3**; **Supplementary Tables 1**, **S2**, **S4**).

### Racial and ethnic disparities

Marked differences in mortality trends were observed across racial and ethnic groups. Hispanic individuals had the highest AAMR at the beginning of the study period, which declined from 4.01 per 100,000 in 1999 to 2.12 per 100,000 in 2023 (AAPC: −2.32; 95% CI: −2.83 to −1.80, P ≤ 0.05). Significant declines were also observed among non-Hispanic White (AAPC: −1.97; 95% CI: −2.15 to −1.78, P ≤ 0.05) and non-Hispanic Other populations (AAPC: −2.31; 95% CI: −2.97 to −1.66, P ≤ 0.05).

**Figure 4:**
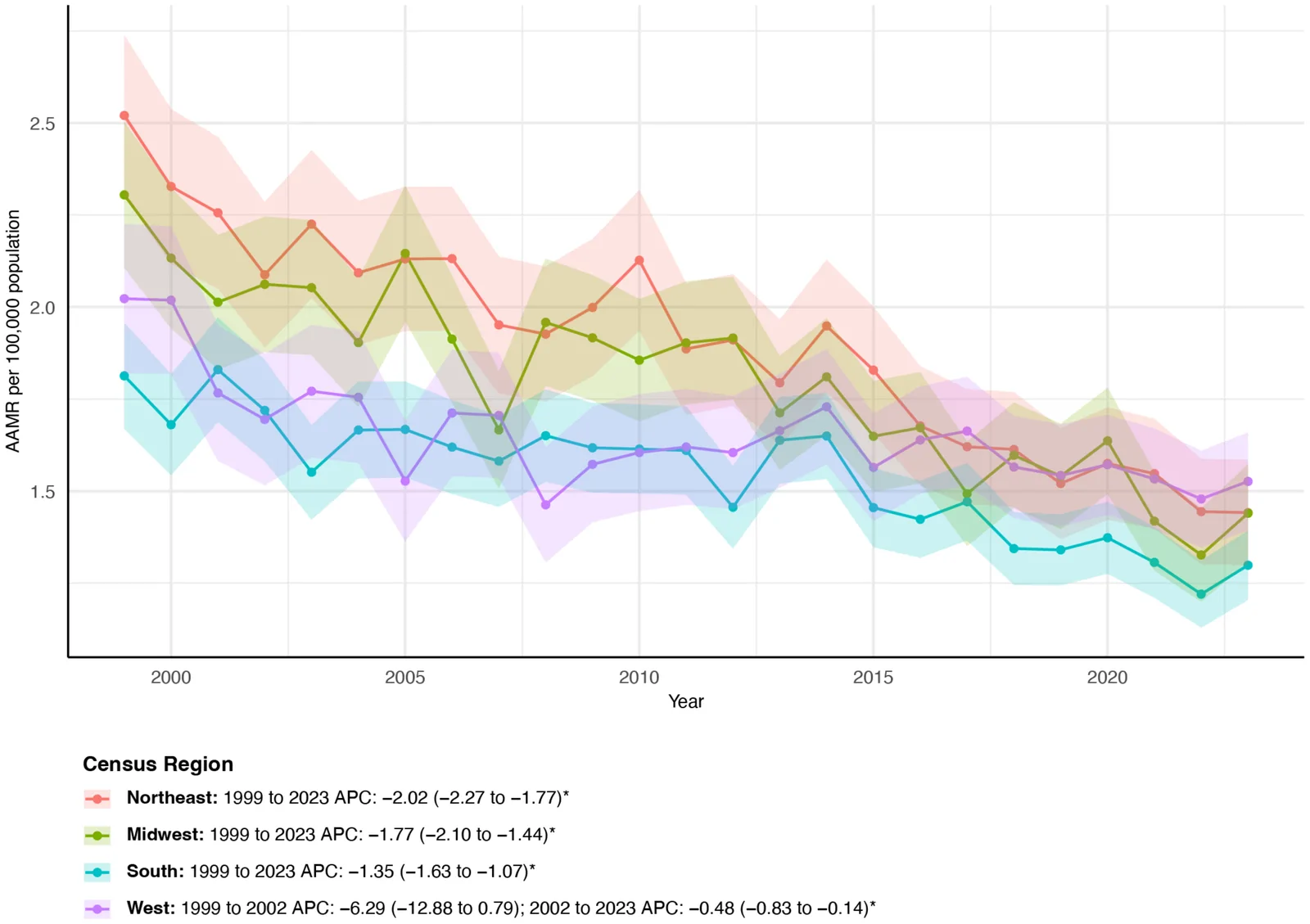
Gallbladder cancer-related age-adjusted mortality rates per 100,000, stratified by race in middle-aged and older adults in the United States, 1999–2023. *Indicates that the annual percentage change (APC) is significantly different from zero at α = 0.05. NH, non-Hispanic.

In contrast, no statistically significant decline in mortality was observed among non-Hispanic Black individuals (AAPC: −0.14; 95% CI: −0.60 to 0.33, P > 0.05) compared with other racial and ethnic groups (**Figure 4**; **Supplementary Tables 2**, **S5**).

### Urbanization-based differences

**Figure 5:**
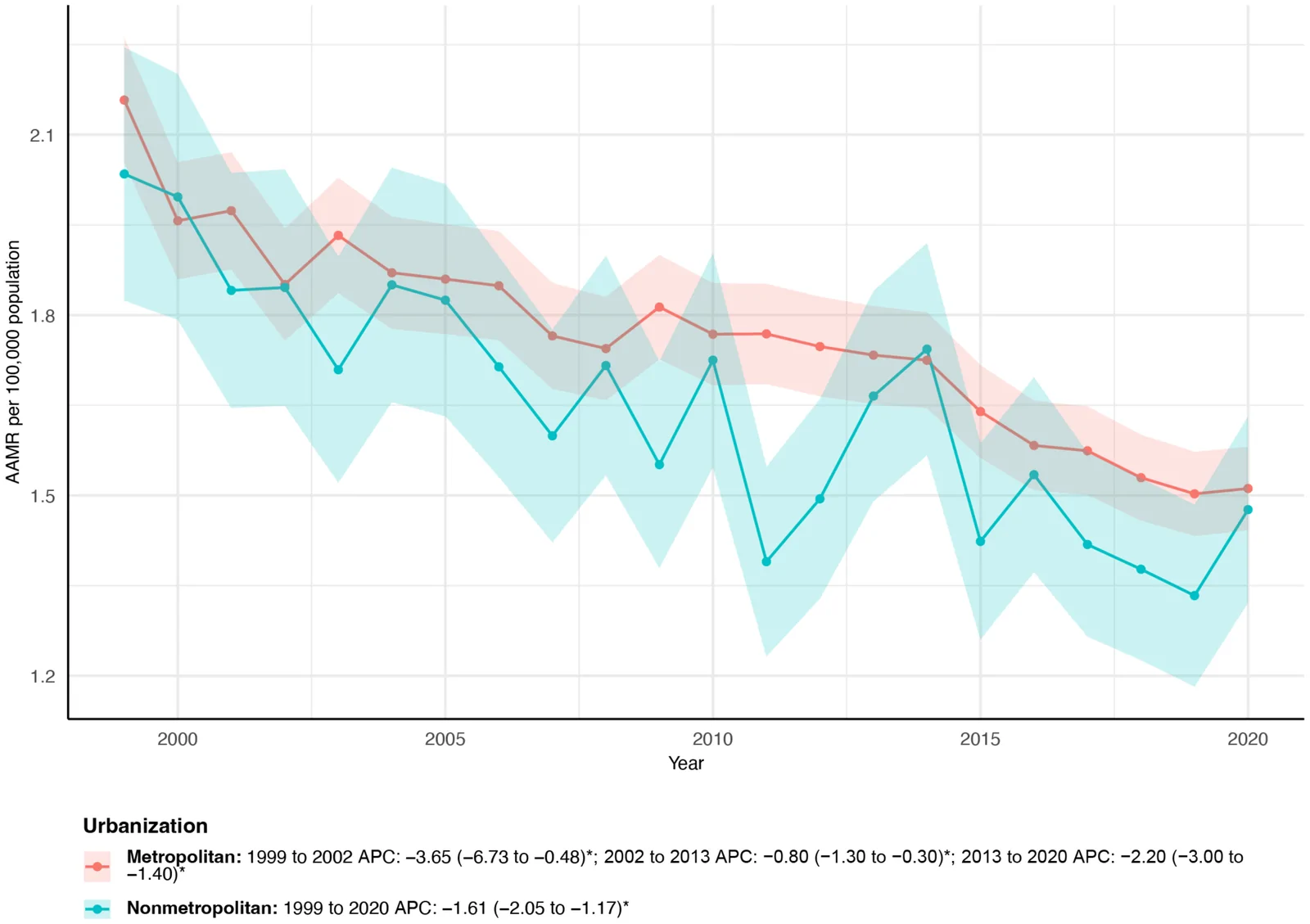
Gallbladder cancer-related age-adjusted mortality rates per 100,000, stratified by urbanization in middle-aged and older adults in the United States, 1999–2020.*Indicates that the annual percentage change (APC) is significantly different from zero at α = 0.05.

For the analysis based on urbanization levels (restricted to the 1999–2020 period), gallbladder cancer mortality was consistently higher in metropolitan areas than in non-metropolitan areas (1.74 vs. 1.63 per 100,000). In metropolitan areas, AAMRs declined significantly across three distinct intervals: from 1999 to 2002 (APC: −3.65; 95% CI: −6.73 to −0.48, P ≤ 0.05), from 2002 to 2013 (APC: −0.80; 95% CI: −1.30 to −0.30, P ≤ 0.05), and from 2013 to 2020 (APC: −2.20; 95% CI: −3.00 to −1.40, P ≤ 0.05). In non-metropolitan areas, mortality also declined significantly over the same period, although with greater year-to-year variability (APC: −1.61; 95% CI: −2.05 to −1.17, P ≤ 0.05) (**Figure 5**; **Supplementary Tables 2**, **S6**).

### Regional variation

Substantial regional differences in gallbladder cancer mortality were observed. The Northeast region consistently demonstrated the highest AAMRs, which declined from 2.52 per 100,000 in 1999 to 1.44 per 100,000 in 2023 (APC: −2.02; 95% CI: −2.27 to −1.77, P ≤ 0.05). Similar downward trends were observed in the Midwest, where mortality declined from 2.30 to 1.44 per 100,000 people (APC: −1.77; 95% CI: −2.10 to −1.44, P ≤ 0.05).

**Figure 6:**
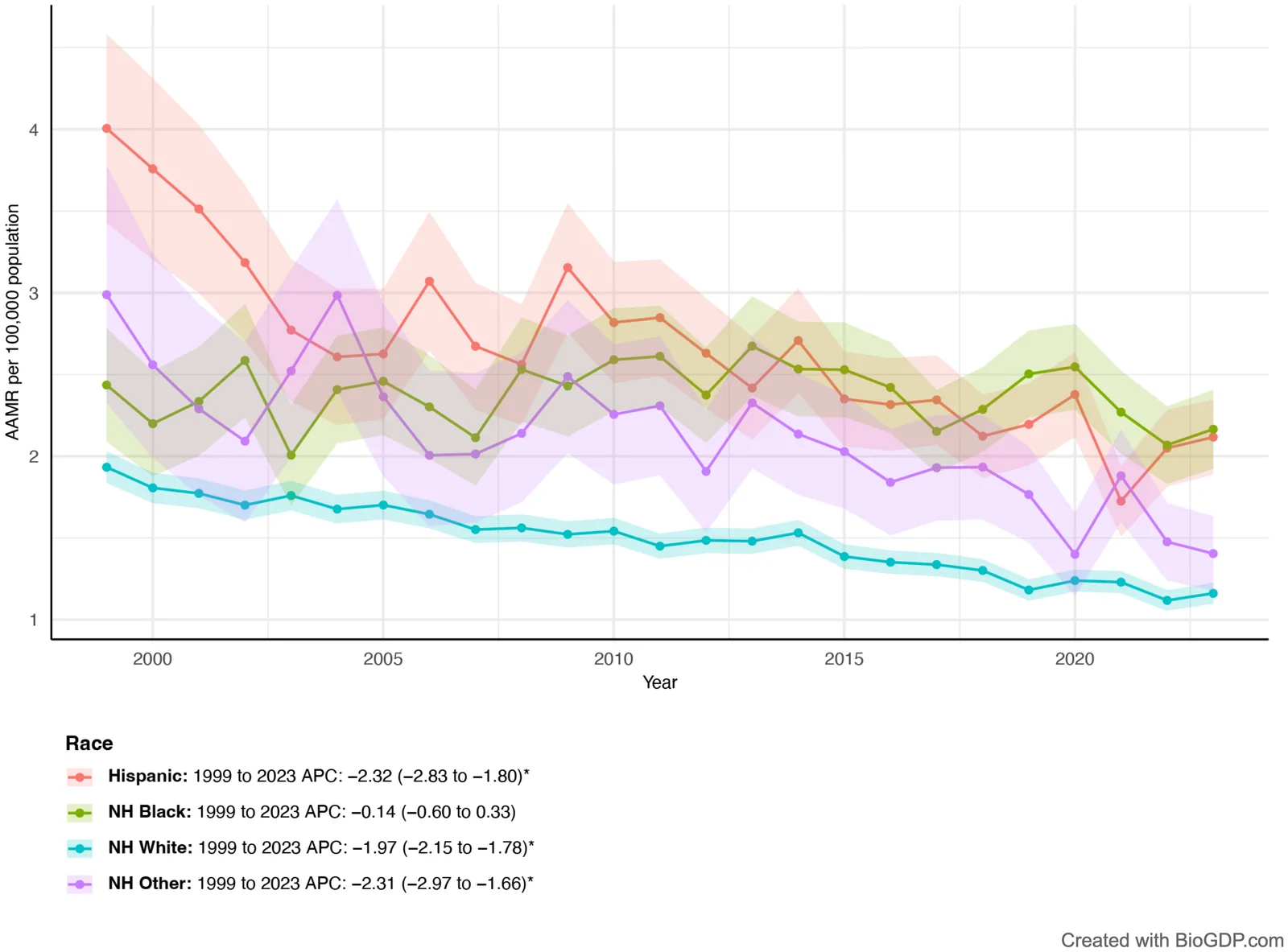
Gallbladder cancer-related age-adjusted mortality rates per 100,000 stratified by census region in middle-aged and older adults in the United States, 1999–2023. *Indicates that the annual percentage change (APC) is significantly different from zero at α = 0.05.

The South region maintained the lowest mortality rates throughout the study period, decreasing from 1.81 per 100,000 in 1999 to 1.30 per 100,000 in 2023 (APC: −1.35; 95% CI: −1.63 to −1.07, P ≤ 0.05). In the West, mortality declined sharply from 1999 to 2002 (APC: −6.29; 95% CI: −12.88 to 0.79, P > 0.05) and subsequently decreased at a slower but statistically significant rate through 2023 (APC: −0.48; 95% CI: −0.83 to −0.14, P ≤ 0.05) (**Figure 6**; **Supplementary Tables 2**, **S7**).

## Discussion

This nationwide analysis provides a comprehensive overview of long-term trends in gallbladder cancer (GBC) mortality among middle-aged and older adults in the United States from 1999 to 2023. Overall, GBC-related mortality declined steadily over the 25-year study period. However, this favorable trend was not uniform across demographic and geographic subgroups. Substantial disparities persisted by sex, age, race and ethnicity, region, and urbanization level, underscoring the uneven distribution of progress in reducing GBC-related mortality.

We hypothesize that the sustained decline in overall age-adjusted mortality might be associated with broader improvements in prevention and clinical management, although this cannot be directly inferred from our mortality data. The widespread use of cholecystectomy for symptomatic gallstone disease has been suggested by previous literature as a potential contributing factor, as most GBC cases are incidentally detected during gallbladder removal (14–16). Since the early 2000s, cholecystectomy has remained one of the most frequently performed abdominal surgeries in the United States, which could theoretically reduce the pool of undiagnosed precursor lesions (17, 18). In addition, it is plausible that advances in surgical techniques and perioperative care, along with the increasing use of adjuvant therapies, could have contributed to improved survival among patients diagnosed at more advanced stages (19–22).

Sex-specific differences in mortality were evident throughout the study period. Women consistently exhibited higher mortality rates than men, which aligns with their higher baseline risk of GBC and the greater prevalence of gallstones among females (23). Hormonal influences on bile composition are thought to play a key role in this disparity. Notably, however, mortality declined more rapidly among women than men. We speculate that this pattern might reflect greater engagement with preventive health services among women, including more frequent abdominal imaging and earlier surgical intervention. Among men, the acceleration in mortality declines after 2013 could potentially be partially attributable to improved control of metabolic risk factors and changes in lifestyle behaviors. Nevertheless, without patient-level behavioral data, these associations warrant further investigation (24).

Age-stratified analyses demonstrated a strong gradient, with mortality increasing markedly with advancing age. Individuals aged 85 years and older consistently experienced the highest mortality rates, reflecting cumulative exposure to chronic inflammation, comorbid conditions, and reduced physiologic reserve. Although mortality declined across all age groups, the persistence of high rates among the oldest adults highlights the need for improved risk stratification and tailored clinical management in aging populations.

Racial and ethnic disparities were among the most striking findings. While Hispanic individuals exhibited the highest mortality rates at baseline, they also experienced the most pronounced declines over time. In contrast, no statistically significant decline in mortality was observed among non-Hispanic Black individuals. This stagnation is deeply concerning and warrants a more comprehensive examination. While our dataset cannot confirm causality, previous literature suggests that the persistent mortality burden in this population is likely multifactorial, potentially driven by intersecting socioeconomic disparities and structural inequities that impede access to high-quality healthcare. Previous research has documented that non-Hispanic Black patients often face systemic barriers, including lower rates of comprehensive health insurance and limited access to primary and specialized care (25–28). Consequently, they are more frequently diagnosed at advanced, unresectable stages of gallbladder cancer compared to their White counterparts (29, 30).

Furthermore, profound disparities in treatment utilization contribute to these mortality patterns. Studies indicate that even when diagnosed at operable stages, Black patients are less likely to undergo definitive surgical resection or receive comprehensive multidisciplinary cancer care (31). These clinical disparities are often compounded by a higher prevalence of metabolic risk factors and broader structural racism, which can manifest as implicit bias and unequal allocation of healthcare resources. Together, we hypothesize that these factors might create a cumulative disadvantage that could partially explain the lack of mortality improvement. However, individual-level socio-demographic and clinical data are required to substantiate these hypotheses.

Differences by urbanization further illustrate potential inequities in healthcare access. Mortality rates were consistently higher in metropolitan areas. Interestingly, while obesity—a major risk factor for GBC—is generally more prevalent in rural U.S. populations, we hypothesize that the higher mortality observed in metropolitan areas may speculatively be driven by other factors (32–34). For instance, metropolitan areas often have higher concentrations of high-risk racial and ethnic populations, such as Hispanic communities, which might contribute to the elevated rates (35). Additionally, this difference might reflect variations in diagnostic accuracy; urban centers with advanced imaging and specialized pathology may more accurately detect and report GBC on death certificates compared to rural settings. Conversely, the more stable and sustained decline observed in metropolitan settings could speculatively be linked to greater access to specialized hepatobiliary centers and multidisciplinary cancer care (36, 37). In contrast, the greater variability observed in non-metropolitan areas may reflect limitations in healthcare infrastructure and workforce shortages, particularly the scarcity of general and specialty surgeons in rural regions (38–40). It is important to reiterate that these explanations are speculative and hypothesis-generating; further studies incorporating direct assessments of these clinical and socioeconomic variables are required to establish causal relationships.

Regional variation in mortality patterns also emerged. The Northeast had the highest AAMRs. Because age-adjusted rates account for differences in population age structure, this elevated mortality cannot be attributed to population aging. Instead, we speculate that it might be driven by regional variations in other risk factors, such as a higher prevalence of chronic liver disease—a known risk factor for GBC or specific environmental and demographic characteristics unique to the region (41). The South consistently demonstrated the lowest mortality rates, possibly reflecting differences in dietary patterns or gallstone prevalence. In the West, the initial rapid decline followed by a slower decrease in mortality may be influenced by the region’s large Hispanic population, which combines higher baseline risk with significant improvements over time (42).

Several limitations should be acknowledged. First, this study relied on death certificate data from the CDC WONDER database, which may be subject to misclassification of cause of death, particularly between gallbladder cancer and other biliary tract malignancies. Second, the lack of individual-level clinical and behavioral data precluded adjustment for specific risk factors or comorbidities. Importantly, the database also lacks individual-level socioeconomic variables, such as income, insurance status, educational attainment and specific metrics of healthcare access. The inability to evaluate these crucial social determinants of health limits our capacity for causal inference and means that these unmeasured factors may partially explain the pronounced racial, ethnic and geographic disparities observed in our study. Third, urbanization and regional classifications may mask within-area heterogeneity. Finally, as the analysis was restricted to the United States, findings may not be generalizable to other countries with different healthcare systems and population structures.

In conclusion, although gallbladder cancer mortality among middle-aged and older adults in the United States has declined over the past two decades, substantial disparities persist. Older adults, women, Hispanic populations, non-Hispanic Black individuals, and residents of certain geographic regions remain disproportionately affected. Addressing these inequities will require coordinated public health strategies focused on early detection, equitable access to surgical care, and targeted prevention efforts in high-risk populations.

## Data Availability

The original contributions presented in the study are included in the article/**Supplementary Material**. Further inquiries can be directed to the corresponding authors.

## Supplementary material

Supplementary Table 1 Gallbladder Cancer-related Mortality, Stra5fied by Sex and Age groups in middle-aged and older adults in the United States, 1999 to 2023.

Supplementary Table 2 Annual percent change (APC) of Gallbladder Cancer–related Age-Adjusted Mortality Rates per 100,000 in middle-aged and older adults in the United States, 1999 to 2023.

Supplementary Table 3 Overall and Sex‐Stratified Gallbladder Cancer–related Age-Adjusted Mortality Rates per 100,000 in middle-aged and older adults in the United States from 1999 to 2023.

Supplementary Table 4 Gallbladder Cancer-related Crude Death Rate per 100,000 stratified by Age Groups in middle-aged and older adults in the United States from 1999 to 2023.

Supplementary Table 5 Gallbladder Cancer-related Age-Adjusted Mortality Rates (AAMR) per 100,000 stratified by Race in middle-aged and older adults in the United States from 1999 to 2023.

Supplementary Table 6 Gallbladder Cancer–related Age-Adjusted Mortality Rates per 100,000 in middle-aged and older adults in the Metropolitan and Non-metropolitan areas in the United States, 1999 to 2020.

Supplementary Table 7 Gallbladder Cancer-related Age-Adjusted Mortality Rates (AAMR) per 100,000 stratified by Census region in middle-aged and older adults in the United States from 1999 to 2023.
