# Supplementary material for: Mortality trends of gallbladder cancer in middle-aged and older adults in the United States: a CDC WONDER analysis from 1999 to 2023
Source: Front Oncol. 2026 Aug 12;16:1881189. doi: 10.3389/fonc.2026.1881189 (PMC13506452; doi:10.3389/fonc.2026.1881189)
Supplement: Supplementary file 1 [file DataSheet1.pdf]

Supplementary File

Supplemental Table 1 Gallbladder Carcinoma-related Mortality, Stratified by Sex and Age groups in middle-aged and older adults in the United States, 1999 to 2023.

	Death								
**Year**	**Overall**	**Women**	**Men**	**45-54 years**	**55-64 years**	**65-74 years**	**75-84 years**	**85+ years**	**Population**
1999	2019	1452	567	127	309	552	686	345	95153686
2000	1920	1365	555	150	285	531	638	316	96944389
2001	1921	1364	557	141	272	512	681	315	99781854
2002	1862	1313	549	156	286	507	591	322	102217733
2003	1891	1302	589	150	291	459	663	328	104692428
2004	1895	1313	582	107	314	507	666	301	107138553
2005	1951	1382	569	157	326	494	631	343	109787199
2006	1951	1385	566	151	334	471	640	355	112380379
2007	1878	1302	576	129	316	467	598	368	114894084
2008	1941	1313	628	139	346	522	613	321	117395131
2009	2024	1368	656	132	378	555	603	356	119895863
2010	2061	1388	673	155	371	574	584	377	121757429
2011	2069	1374	695	149	395	558	580	387	124174484
2012	2062	1391	671	125	364	597	598	378	126000296
2013	2131	1392	739	161	384	613	618	355	127788037
2014	2240	1537	703	148	415	658	654	365	129779643
2015	2093	1447	646	155	359	558	617	404	131826832
2016	2135	1441	694	137	372	642	602	382	133494018
2017	2142	1422	720	129	373	642	619	379	135229289
2018	2137	1451	686	131	390	633	614	369	136335528
2019	2117	1423	694	136	404	627	587	363	137381702
2020	2235	1483	752	120	430	685	619	381	138429175
2021	2089	1397	692	117	372	666	600	334	139339453
2022	2040	1355	685	116	335	664	593	332	140311934
2023	2134	1459	675	123	334	683	650	344	141596553
**Total**	50938	34819	16119	3441	8755	14357	15545	8820	3043725672

Supplemental Table 2 Annual percent change (APC) of Gallbladder Carcinoma–related Age-Adjusted Mortality Rates per 100,000 in middle-aged and older adults in the United States, 1999 to 2023

Year Interval	APC (95% CI)
**Overall**	
1999-2002	-3.60 (-6.22 to -0.89) *
2002-2014	-1.02 (-1.38 to -0.65) *
2014-2023	-2.14 (-2.62 to -1.65) *
**Men**	
1999-2013	-0.68 (-1.22 to -0.13) *
2013-2023	-2.39 (-3.23 to -1.54) *
**Women**	
1999-2023	-1.50 (-1.70 to -1.30) *
**45-54 years**	
1999-2023	-0.77 (-1.33 to -0.21) *
**55-64 years**	
1999-2023	-1.27 (-1.62 to -0.91) *
**65-74 years**	
1999-2023	-1.64 (-1.92 to -1.36) *
**75-84 years**	
1999-2015	-1.18 (-1.64 to -0.71) *
2015-2023	-3.04 (-4.36 to -1.71) *
**85+ years**	
1999-2023	-1.58 (-1.84 to -1.32) *
**NH White**	
1999-2023	-1.97 (-2.15 to -1.78) *
**NH Black**	
1999-2023	-0.14 (-0.60 to 0.32)
**Hispanic**	
1999-2023	-2.32 (-2.83 to -1.80) *
**NH Other**	
1999-2023	-2.31 (-2.97 to -1.66) *
**Non-metropolitan areas**	
1999-2020	-1.61 (-2.05 to -1.17) *
**Metropolitan area**	
1999-2002	-3.65 (-6.73 to -0.48) *
2002-2013	-0.80 (-1.30 to -0.30) *
2013-2020	-2.20 (-3.00 to -1.40) *
**Midwest**	
1999-2023	-1.77 (-2.10 to 1.44) *
**Northeast**	
1999-2023	-2.02 (-2.27 to -1.77) *
**South**	
1999-2023	-1.35 (-1.63 to -1.07) *
**West**	
1999-2002	-6.29 (-12.88 to 0.79)
2002-2023	-0.48 (-0.83 to -0.14) *

APC = Annual percent change; NH = non-Hispanic; NH Black = NH Black or African American; NH Other = American Indian or Alaska Native or Asian or Pacific Islander; Hispanic = Hispanic or Latino. The APC for age groups is based on crude mortality rates.

Supplemental Table 3 Overall and Sex-Stratified Gallbladder Carcinoma–related Age-Adjusted Mortality Rates per 100,000 in middle-aged and older adults in the United States from 1999 to 2023

	Age-Adjusted Rate (95% CI)		
**Year**	**Men**	**Women**	**Overall**
1999	1.47 (1.35-1.59)	2.60 (2.47 -2.74)	2.10 (2.01-2.19)
2000	1.41 (1.29-1.53)	2.44 (2.31-2.57)	2.01 (1.92-2.10)
2001	1.39 (1.27-1.50)	2.38 (2.25-2.50)	1.98 (1.89-2.07)
2002	1.36 (1.24-1.47)	2.29 (2.16-2.41)	1.88 (1.80-1.97)
2003	1.39 (1.28-1.51)	2.19 (2.07-2.31)	1.86 (1.78-1.94)
2004	1.37 (1.26-1.49)	2.18 (2.06-2.30)	1.85 (1.77-1.94)
2005	1.28 (1.17-1.39)	2.27 (2.15-2.39)	1.87 (1.78-1.95)
2006	1.26 (1.15-1.36)	2.23 (2.11-2.35)	1.80 (1.72-1.88)
2007	1.27 (1.16-1.37)	2.07 (1.96-2.19)	1.74 (1.66-1.82)
2008	1.32 (1.21-1.42)	2.04 (1.93-2.15)	1.72 (1.64-1.80)
2009	1.35 (1.24-1.45)	2.10 (1.99-2.21)	1.77 (1.69-1.85)
2010	1.35 (1.25-1.46)	2.11 (2.00-2.22)	1.75 (1.67-1.82)
2011	1.34 (1.24-1.44)	2.03 (1.92-2.14)	1.70 (1.63-1.78)
2012	1.25 (1.16-1.35)	1.99 (1.88-2.10)	1.68 (1.61-1.75)
2013	1.36 (1.26-1.46)	2.01 (1.90-2.11)	1.71 (1.64-1.79)
2014	1.22 (1.13-1.32)	2.15 (2.04-2.26)	1.72 (1.65-1.79)
2015	1.13 (1.05-1.22)	1.98 (1.88-2.09)	1.61 (1.54-1.68)
2016	1.20 (1.11-1.29)	1.93 (1.83-2.03)	1.57 (1.50-1.63)
2017	1.19 (1.10-1.28)	1.86 (1.76-1.96)	1.56 (1.49-1.63)
2018	1.08 (1.00-1.16)	1.86 (1.77-1.96)	1.50 (1.44-1.57)
2019	1.08 (0.99-1.16)	1.76 (1.66-1.85)	1.47 (1.40-1.53)
2020	1.17 (1.08-1.25)	1.80 (1.71-1.89)	1.51 (1.44-1.57)
2021	1.04 (0.96-1.12)	1.72 (1.63-1.81)	1.45 (1.38-1.51)
2022	1.01 (0.93-1.09)	1.64 (1.55-1.73)	1.36 (1.30-1.42)
2023	1.01(0.93-1.09)	1.72 (1.63-1.81)	1.39 (1.34-1.45)

Supplemental Table 4 Gallbladder Carcinoma-related Crude Death Rate per 100,000 stratified by Age Groups in middle-aged and older adults in the United States from 1999 to 2023

	Crude Death Rate (95% CI)				
**Year**	**45-54 years**	**55-64 years**	**65-74 years**	**75-84 years**	**85+ years**
1999	0.35 (0.29-0.41)	1.30 (1.15-1.44)	3.00 (2.75-3.25)	5.61 (5.19-6.03)	8.31 (7.43-9.18)
2000	0.40 (0.33-0.46)	1.17 (1.04-1.31)	2.89 (2.64-3.13)	5.16 (4.76-5.56)	7.45 (6.63-8.28)
2001	0.36 (0.30-0.42)	1.08 (0.95-1.21)	2.79 (2.54-3.03)	5.41 (5.00-5.81)	7.30 (6.50-8.11)
2002	0.39 (0.33-0.45)	1.07 (0.95-1.20)	2.76 (2.52-3.00)	4.63 (4.26-5.00)	7.37 (6.57-8.18)
2003	0.37 (0.31-0.43)	1.04 (0.92-1.16)	2.48 (2.25-2.71)	5.14 (4.75-5.53)	7.34 (6.55-8.14)
2004	0.26 (0.21-0.31)	1.07 (0.95-1.19)	2.72 (2.48-2.95)	5.13 (4.74-5.52)	6.62 (5.87-7.37)
2005	0.37 (0.31-0.43)	1.06 (0.95-1.18)	2.61 (2.39-2.85)	4.83 (4.45-5.20)	7.31 (6.53-8.08)
2006	0.35 (0.29-0.40)	1.05 (0.93-1.16)	2.45 (2.23-2.67)	4.89 (4.51-5.27)	7.30 (6.54-8.05)
2007	0.29 (0.24-0.34)	0.95 (0.85-1.06)	2.37 (2.16-2.59)	4.57 (4.20-4.94)	7.30 (6.56-8.05)
2008	0.31 (0.26-0.36)	1.01 (0.91-1.12)	2.55 (2.33-2.76)	4.69 (4.32-5.06)	6.18 (5.50-6.85)
2009	0.29 (0.24-0.34)	1.07 (0.96-1.18)	2.61 (2.40-2.83)	4.63 (4.26-5.00)	6.63 (5.94-7.32)
2010	0.34 (0.29-0.40)	1.02 (0.91-1.12)	2.64 (2.43-2.86)	4.47 (4.11-4.83)	6.86 (6.17-7.56)
2011	0.33 (0.28-0.39)	1.04 (0.94-1.14)	2.48 (2.28-2.69)	4.40 (4.04-4.76)	6.75 (6.07-7.42)
2012	0.28 (0.23-0.33)	0.94 (0.85-1.04)	2.49 (2.29-2.69)	4.51 (4.14-4.87)	6.42 (5.77-7.07)
2013	0.37 (0.31-0.42)	0.98 (0.88-1.07)	2.43 (2.24-2.62)	4.60 (4.23-4.96)	5.88 (5.27-6.49)
2014	0.34 (0.29-0.40)	1.04 (0.94-1.14)	2.49 (2.30-2.68)	4.78 (4.41-5.15)	5.92 (5.32-6.53)
2015	0.36 (0.30-0.42)	0.88 (0.79-0.97)	2.03 (1.86-2.19)	4.43 (4.08-4.78)	6.43 (5.80-7.05)
2016	0.32 (0.27-0.37)	0.90 (0.81-0.99)	2.24 (2.07-2.42)	4.23 (3.89-4.57)	5.99 (5.39-6.59)
2017	0.30 (0.25-0.36)	0.89 (0.80-0.98)	2.16 (2.00-2.33)	4.21 (3.88-4.54)	5.86 (5.27-6.45)
2018	0.31 (0.26-0.37)	0.92 (0.83-1.01)	2.08 (1.91-2.24)	3.99 (3.67-4.30)	5.63 (5.06-6.21)
2019	0.33 (0.28-0.39)	0.95 (0.86-1.04)	1.99 (1.84-2.15)	3.68 (3.38-3.97)	5.50 (4.93-6.06)
2020	0.30 (0.24-0.35)	1.01 (0.92-1.11)	2.10 (1.95-2.26)	3.76 (3.47-4.06)	5.72 (5.15-6.30)
2021	0.29 (0.24-0.34)	0.87 (0.78-0.96)	1.98 (1.83-2.13)	3.70 (3.41-4.00)	5.59 (4.99-6.19)
2022	0.29 (0.23-0.34)	0.80 (0.71-0.88)	1.97 (1.82-2.11)	3.38 (3.11-3.66)	5.12 (4.57-5.67)
2023	0.30 (0.25-0.36)	0.80 (0.71-0.88)	1.97 (1.82-2.12)	3.54 (3.27-3.81)	5.55 (4.97-6.14)

Supplemental Table 5 Gallbladder Carcinoma-related Age-Adjusted Mortality Rates (AAMR) per 100,000 stratified by Race in middle-aged and older adults in the United States from 1999 to 2023

	Age-Adjusted Rate (95% CI)			
**Year**	**NH White**	**NH Black**	**Hispanic**	**NH Other**
1999	1.93 (1.84-2.03)	2.44 (2.09-2.78)	4.01 (3.43-4.58)	2.99 (2.33-3.78)
2000	1.81 (1.71-1.90)	2.20 (1.88-2.52)	3.76 (3.20-4.31)	2.56 (1.99-3.24)
2001	1.77 (1.68-1.86)	2.34 (2.00-2.67)	3.51 (3.00-4.03)	2.29 (1.76-2.93)
2002	1.70 (1.61-1.79)	2.59 (2.24-2.93)	3.18 (2.71-3.66)	2.09 (1.59-2.70)
2003	1.76 (1.67-1.85)	2.01 (1.70-2.31)	2.77 (2.34-3.21)	2.52 (2.00-3.14)
2004	1.68 (1.59-1.76)	2.41 (2.08-2.74)	2.61 (2.19-3.03)	2.99 (2.40-3.57)
2005	1.70 (1.61-1.79)	2.46 (2.13-2.79)	2.63 (2.23-3.02)	2.36 (1.88-2.93)
2006	1.64 (1.56-1.73)	2.30 (1.99-2.62)	3.07 (2.65-3.50)	2.01 (1.57-2.52)
2007	1.55 (1.47-1.63)	2.11 (1.82-2.41)	2.67 (2.28-3.06)	2.01 (1.59-2.51)
2008	1.56 (1.48-1.64)	2.53 (2.21-2.85)	2.56 (2.19-2.93)	2.14 (1.72-2.63)
2009	1.52 (1.44-1.60)	2.43 (2.12-2.74)	3.15 (2.76-3.55)	2.49 (2.02-2.96)
2010	1.54 (1.46-1.62)	2.59 (2.27-2.91)	2.82 (2.45-3.19)	2.26 (1.83-2.68)
2011	1.45 (1.37-1.53)	2.61 (2.30-2.92)	2.85 (2.49-3.20)	2.31 (1.88-2.73)
2012	1.48 (1.41-1.56)	2.37 (2.08-2.67)	2.63 (2.29-2.97)	1.91 (1.53-2.28)
2013	1.48 (1.40-1.56)	2.67 (2.37-2.98)	2.42 (2.10-2.73)	2.33 (1.93-2.73)
2014	1.53 (1.45-1.61)	2.53 (2.24-2.82)	2.71 (2.39-3.03)	2.14 (1.76-2.51)
2015	1.39 (1.31-1.46)	2.53 (2.24-2.82)	2.35 (2.06-2.64)	2.03 (1.68-2.38)
2016	1.35 (1.28-1.42)	2.42 (2.14-2.70)	2.32 (2.03-2.60)	1.84 (1.52-2.17)
2017	1.34 (1.27-1.41)	2.15 (1.90-2.41)	2.34 (2.07-2.62)	1.93 (1.61-2.25)
2018	1.30 (1.23-1.37)	2.29 (2.03-2.55)	2.12 (1.87-2.38)	1.93 (1.61-2.25)
2019	1.18 (1.12-1.25)	2.50 (2.24-2.77)	2.19 (1.94-2.45)	1.77 (1.47-2.06)
2020	1.24 (1.17-1.30)	2.55 (2.28-2.81)	2.38 (2.12-2.64)	1.40 (1.15-1.65)
2021	1.23 (1.16-1.30)	2.27 (2.02-2.52)	1.73 (1.51-1.94)	1.88 (1.60-2.16)
2022	1.12 (1.06-1.18)	2.07 (1.83-2.31)	2.05 (1.81-2.28)	1.48 (1.24-1.71)
2023	1.16 (1.10-1.22)	2.17 (1.92-2.41)	2.12 (1.89-2.35)	1.40 (1.18-1.63)

Supplemental Table 6 Gallbladder Carcinoma–related Age-Adjusted Mortality Rates per 100,000 in middle-aged and older adults in the Metropolitan and Non-metropolitan areas in the United States, 1999 to 2020

	**Age-Adjusted Rate (95% CI)**	
**Year**	**Metropolitan**	**Nonmetropolitan**
1999	2.16 (2.05-2.26)	2.03 (1.82-2.25)
2000	1.96 (1.86-2.05)	2.00 (1.79-2.20)
2001	1.97 (1.88-2.07)	1.84 (1.65-2.04)
2002	1.85 (1.76-1.94)	1.85 (1.65-2.04)
2003	1.93 (1.84-2.03)	1.71 (1.52-1.90)
2004	1.87 (1.78-1.96)	1.85 (1.66-2.05)
2005	1.86 (1.77-1.95)	1.82 (1.63-2.02)
2006	1.85 (1.76-1.94)	1.71 (1.53-1.90)
2007	1.77 (1.68-1.85)	1.60 (1.42-1.78)
2008	1.74 (1.66-1.83)	1.72 (1.53-1.90)
2009	1.81 (1.73-1.90)	1.55 (1.38-1.72)
2010	1.77 (1.68-1.85)	1.73 (1.55-1.90)
2011	1.77 (1.69-1.85)	1.39 (1.23-1.55)
2012	1.75 (1.66-1.83)	1.49 (1.33-1.66)
2013	1.73 (1.65-1.81)	1.67 (1.49-1.84)
2014	1.72 (1.65-1.80)	1.74 (1.57-1.92)
2015	1.64 (1.56-1.72)	1.42 (1.26-1.59)
2016	1.58 (1.51-1.66)	1.53 (1.37-1.70)
2017	1.57 (1.50-1.65)	1.42 (1.27-1.57)
2018	1.53 (1.46-1.60)	1.38 (1.23-1.53)
2019	1.50 (1.43-1.57)	1.33 (1.18-1.48)
2020	1.51 (1.44-1.58)	1.48 (1.32-1.63)
**Overall**	1.74 (1.72-1.76)	1.63 (1.59-1.67)

Supplemental Table 7 Gallbladder Carcinoma-related Age-Adjusted Mortality Rates (AAMR) per 100,000 stratified by Census region in middle-aged and older adults in the United States from 1999 to 202

	Age-Adjusted Rate (95% CI)			
**Year**	**Northeast**	**Midwest**	**South**	**West**
1999	2.52 (2.30-2.74)	2.30 (2.11-2.50)	1.81 (1.67-1.96)	2.02 (1.82-2.23)
2000	2.33 (2.12-2.54)	2.13 (1.94-2.32)	1.68 (1.54-1.82)	2.02 (1.82-2.22)
2001	2.26 (2.05-2.46)	2.01 (1.83-2.20)	1.83 (1.69-1.97)	1.77 (1.58-1.95)
2002	2.09 (1.89-2.28)	2.06 (1.88-2.25)	1.72 (1.58-1.86)	1.69 (1.52-1.87)
2003	2.23 (2.02-2.43)	2.05 (1.87-2.24)	1.55 (1.42-1.68)	1.77 (1.59-1.95)
2004	2.09 (1.90-2.29)	1.90 (1.73-2.08)	1.67 (1.53-1.80)	1.75 (1.58-1.93)
2005	2.13 (1.93-2.33)	2.15 (1.96-2.33)	1.67 (1.54-1.80)	1.53 (1.36-1.69)
2006	2.13 (1.94-2.33)	1.91 (1.74-2.09)	1.62 (1.49-1.75)	1.71 (1.54-1.88)
2007	1.95 (1.77-2.14)	1.67 (1.51-1.83)	1.58 (1.46-1.71)	1.71 (1.54-1.87)
2008	1.93 (1.74-2.11)	1.96 (1.79-2.13)	1.65 (1.53-1.78)	1.46 (1.31-1.62)
2009	2.00 (1.81-2.18)	1.92 (1.75-2.09)	1.62 (1.50-1.74)	1.57 (1.42-1.73)
2010	2.13 (1.94-2.32)	1.86 (1.69-2.02)	1.61 (1.49-1.74)	1.60 (1.45-1.76)
2011	1.89 (1.71-2.06)	1.90 (1.74-2.07)	1.61 (1.49-1.73)	1.62 (1.46-1.78)
2012	1.91 (1.73-2.09)	1.92 (1.75-2.08)	1.46 (1.34-1.57)	1.60 (1.45-1.76)
2013	1.79 (1.62-1.97)	1.71 (1.56-1.87)	1.64 (1.52-1.76)	1.66 (1.51-1.82)
2014	1.95 (1.77-2.13)	1.81 (1.65-1.97)	1.65 (1.53-1.77)	1.73 (1.57-1.89)
2015	1.83 (1.66-2.00)	1.65 (1.50-1.80)	1.45 (1.35-1.56)	1.56 (1.42-1.71)
2016	1.68 (1.51-1.84)	1.67 (1.52-1.82)	1.42 (1.32-1.53)	1.64 (1.49-1.78)
2017	1.62 (1.46-1.78)	1.49 (1.35-1.63)	1.47 (1.37-1.58)	1.66 (1.52-1.81)
2018	1.61 (1.46-1.77)	1.60 (1.45-1.74)	1.34 (1.24-1.44)	1.57 (1.43-1.70)
2019	1.52 (1.37-1.67)	1.54 (1.40-1.68)	1.34 (1.24-1.44)	1.54 (1.40-1.68)
2020	1.57 (1.42-1.73)	1.64 (1.49-1.78)	1.37 (1.28-1.47)	1.57 (1.44-1.71)
2021	1.55 (1.40-1.70)	1.42 (1.28-1.55)	1.31 (1.21-1.40)	1.53 (1.40-1.67)
2022	1.44 (1.30-1.59)	1.33 (1.20-1.45)	1.22 (1.13-1.31)	1.48 (1.35-1.61)
2023	1.44 (1.30-1.58)	1.44 (1.31-1.57)	1.30 (1.20-1.39)	1.53 (1.39-1.66)
